# Directed Modification of a GHF11 Thermostable Xylanase AusM for Enhancing Inhibitory Resistance towards SyXIP-I and Application of AusM^PKK^ in Bread Making

**DOI:** 10.3390/foods12193574

**Published:** 2023-09-26

**Authors:** Dong Zhang, Jing Huang, Youyi Liu, Xingyi Chen, Tiecheng Gao, Ning Li, Weining Huang, Minchen Wu

**Affiliations:** 1 Wuxi School of Medicine, Jiangnan University, Wuxi 214122, China; 2 Key Laboratory of Carbohydrate Chemistry and Biotechnology, Ministry of Education, School of Biotechnology, Jiangnan University, Wuxi 214122, China; 3 State Key Laboratory of Food Science and Technology, and the Laboratory of Baking and Fermentation Science, Cereals/Sourdough and Nutritional Functionality Research, School of Food Science and Technology, Jiangnan University, Wuxi 214122, China; 4 Guangzhou Puratos Food Co., Ltd., Guangzhou 511400, China

**Keywords:** arabinoxylan, xylanase, inhibition sensitivity, computer-aided re-design, bread baking

## Abstract

To reduce the inhibition sensitivity of a thermoresistant xylanase AusM to xylanase inhibitor protein (XIP)-type in wheat flour, the site-directed mutagenesis was conducted based on the computer-aided redesign. First, fourteen single-site variants and one three-amino acid replacement variant in the thumb region of an AusM-encoding gene (*AusM*) were constructed and expressed in *E. coli* BL21(DE3), respectively, as predicted theoretically. At a molar ratio of 100:1 between SyXIP-I/xylanase, the majority of mutants were nearly completely inactivated by the inhibitor SyXIP-I, whereas AusM^N127A^ retained 62.7% of its initial activity and AusM^PKK^ retained 100% of its initial activity. The optimal temperature of the best mutant AusM^PKK^ was 60 °C, as opposed to 60–65 °C for AusM, while it exhibited improved thermostability, retaining approximately 60% of its residual activity after heating at 80 °C for 60 min. Furthermore, AusM^PKK^ at a dosage of 1000 U/kg was more effective than AusM at 4000 U/kg in increasing specific bread loaf volume and reducing hardness during bread production and storage. Directed evolution of AusM significantly reduces inhibition sensitivity, and the mutant enzyme AusM^PKK^ is conducive to improving bread quality and extending its shelf life.

## 1. Introduction

Arabinoxylan (AX), a major non-starch carbohydrate, is present in wheat flour at relatively low concentrations of 1.5−3.0% *w*/*w* dm. It comprises a linear framework of β-1,4-linked D-xylose units where arabinose residues can be added at the O-2 and/or O-3 positions [1,2]. Ferulic and *p*-coumaric acids can form ester bonds with the arabinose side chains of AX [3]. AX, a substance with the ability to bind to water at a ratio of nearly ten times its weight, equivalent to about 30% of the water-binding capacity in wheat flour, had the potential to influence the formation of the gluten network by competing for water with gluten and starch during the bread-making process, resulting in low volume and high hardness of bread [4,5]. According to their physical and chemical properties, the AXs in wheat flour can be divided into two types: one is water-unextractable (WU-AX) (70−75%), and another one is water-extractable (WE-AX) (25−30%) [6]. WU-AX is structurally very similar to WE-AX, whereas it has a slightly higher average molecular weight and arabinose to xylose ratio (A/X) than WE-AX [7]. According to research by Courtin, WU-AX has several negative effects on baking quality. In contrast, WE-AX and solubilized AX(S-AX) with medium to high molecular weight have been found to exert beneficial effects [8].

Enzymes are commonly added to flour during baking to mitigate the adverse impacts of arabinoxylans (AX) and improve the overall bread quality [9]. An example of an enzyme (endo-β-1,4-D-xylanase, EC 3.2.1.8), which exhibits the ability to cleave the β-1,4-xylosidic linkages in the main chain of AX, thereby altering its functional and physicochemical properties [10]. Based on their amino acid sequence similarities, xylanases are primarily classified into glycoside hydrolase families(GHF) 10 and 11 [11]. The two GHF xylanases exhibit variations in their three-dimensional structures, molecular weights, and catalytic properties. In contrast to GHF10 xylanases, GHF11 xylanases are regarded as genuine, primarily due to their notable substrate specificity [12]. Additionally, GHF11 xylanases prefer to hydrolyze WU-AX while retaining WE-AX and S-AX, which had a positive impact on bread loaf volume [13]. GHF11 xylanases can be obtained from diverse sources, such as bacteria and fungi, which facilitates their application [14].

Xylanase inhibitors affect xylanase activity in bread making. These inhibitors can potentially modify the substrate selectivity of xylanases by exhibiting a preference for binding to WU-AX rather than WE-AX. This disrupted the balance between the process of enzymatic solubilization of AX and the depolymerization of AX [15]. To date, three distinct proteinaceous xylanase inhibitors have been identified in cereals. These inhibitors, known as the TAXI (Triticum aestivum xylanase inhibitor)-type [16], the XIP (xylanase inhibitor protein)-type [17] and the TLXI (thaumatin-like xylanase inhibitor)-type inhibitors [18], exhibit different structures and specificities. The TAXI, XIP, and TLXI inhibitor protein levels in eight wheat cultivars were quantified using immunoblotting, yielding average concentrations of 133, 235, and 112 ppm, respectively [19]. The inhibitory activity of these inhibitors exhibits variation contingent upon the source and type of the validated xylanase. Both TAXI and TLXI inhibitors have been found to inhibit GHF11 xylanases from fungal and bacterial origins but not GHF10 xylanases, while XIP-type inhibitors mostly inhibit GHF11 and GHF10 xylanases from fungal sources [20,21]. Additionally, it has been demonstrated that XIP-I can effectively suppress the enzymatic function of both AMY1 and AMY2 (barley-amylase ioszymes) [22].

*AuXyn11A* (GeneBank: HQ724284.1), a gene encoding AuXyn11A, was cloned from *Aspergillus usamii* E001 and successfully expressed in *Pichia pastoris* GS115 in our previous research. AusM, a hybrid mutant with high specific activity and improved thermostability, was then obtained by N-terminal region fragment substitution using rational design. This research aims to create mutants with high thermostability and enhanced resistance to SyXIP-I for use in bread production. Using the seamless cloning technique, the *AusM* gene from pUCm-T/*AusM* was cloned into the pET-28a(+) plasmid and expressed in *E. coli* BL21(DE3). To improve the insensitivity of AusM to SyXIP-I, fifteen variants of AusM were constructed by two-step PCR based on a computer-aided redesign and expressed in *E. coli*. Both thermostability and insensitivity to SyXIP-I were determined, respectively, and compared with those of AusM. Furthermore, both AusM and AusM^PKK^ were purified and used in bread baking, which can significantly increase specific bread loaf volume and decrease the hardness of bread during storage, effectively extending the shelf-life of the bread and mitigating the aging process.

## 2. Materials and Methods

### 2.1. Strains, Plasmids and Primers

Both *E. coli* BL21(DE3) and *P. pastoris* X33 (Novagen, Madison, WI, USA) were used as the hosts for the expression of recombinant proteins. The plasmids pET-28a(+) and pGAPZαA (Takara, Dalian, China) with kanamycin and zeocin resistance, respectively, were used as the expression vectors. The recombinant plasmids, namely, pUCm-T/*AusM* and pGAPZαA/*Syxip*-I, were constructed and preserved in our laboratory. Primers synthesized by Yixin Biotech. Co., Ltd. (Wuxi, China), were listed in Table 1.

### 2.2. Chemicals

Birchwood xylan and IPTG were purchased from Sigma-Aldrich (St. Louis, MO, USA). Gu’an Shenhua Flour Co., Ltd. (Langfang, China) supplied us with commercial wheat flour comprising 13.7% protein, 1.3% crude fat, and 70.9% starch. All other chemical reagents used in the study were of analytical grade and obtained from commercial sources (Wuxi, China). Hieff Canace^®^ Plus PCR Master Mix and Hieff Clone^®^ Universal One Step Cloning Kit were purchased from Yeasen Biotech Co., Ltd. (Shanghai, China).

### 2.3. Homology Modeling and Computer-Aided Re-Design of AusM for Its Site-Directed Mutagenesis

Using AusM as a template, several typical families of 11 xylanases with more than 70% sequence identity with AusM were searched by a BLAST server at the NCBI website (http://www.ncbi.nlm.nih.gov/, accessed on 1 August 2023), among which four enzymes (*Ak*XYNC, *An*XYNA, *Tr*XYN1, *Tl*xynA) [23,24,25,26] with inhibitor sensitivity and one (*Pg*xynB) [27] with significant insensitivity were ultimately chosen to conduct the multiple sequence alignment with AusM. The 3-D structures of AusM and its mutant AusM^PKK^ were homologically modeled using the MODELLER 9.11 program (http://salilab.org/modeller/, accessed on 5 August 2023) based on the crystal structure of a thermostable mutant of GHF11 xylanase that was isolated from the environment and had a known crystal structure at a resolution of 1.90 Å (PDB: 2vu1). The mutant shares 74.09% identity with AusM. Meanwhile, the 3-D structure of xylanase inhibitor protein SyXIP-I came from the crystal structure of xylanase inhibitor protein (XIP-I) from wheat at 1.80 Å resolution (PDB: 1om0), sharing 100% identity with SyXIP-I. To reduce its inhibitor sensitivity towards SyXIP-I, the mutual action between the 3-D structures of AusM and SyXIP-I was predicted by Z-dock using Discovery Studio 2019 to locate the most appropriate binding sites. Z-dock was conducted according to the protocol of Discovery Studio 2019.The final docked conformation was chosen from the docking process, having the best Z-dock, Z-rank, and R-dock scores among all the refined conformations. Based on the optimized complex, the residues of AusM in close proximity to a π loop of SyXIP-I were identified using a PyMol software (http://pymol.org/, Version 1.1).

The multiple sequence alignment of AusM with the other five GHF11 xylanases was carried out using the ClustalW2 program (http://www.ebi.ac.uk/Tools/msa/clustalw2/, accessed on 10 August 2023). The above-identified amino acids of AusM, whose frequencies were above 80% among six xylanases, were considered conserved, while those frequencies were below 60%, and those that appeared in the resistant xylanase were deemed non-conserved to this work. Several residues were selected to be substituted with a smaller size Alanine or the corresponding ones among the resistant xylanase, respectively, to construct a series of mutants of AusM.

### 2.4. Cloning, Mutagenesis and Protein Expression

A 6 × His-tag was added at the C-terminus of the xylanase gene in the recombinant plasmid. The AusM coding sequence was amplified by PCR from the pUCm-T-*AusM* plasmid [28] using AusM-F and AusM-R primers (Table 1). Additionally, using pET-28a-*AusM* as a template, reverse PCR primers r-pET-28a-F and r-pET-28a-R were used to generate the linearized vector. The xylanase gene was then inserted into the proper location of the plasmid pET-28a(+) by Hieff Clone^®^ Universal One Step Cloning Kit. The recombinant plasmid pET-28a-*AusM* was subsequently transformed into *E. coli* BL21(DE3). The positive clones of *E. coli*/*AusM* were verified by nucleotide sequence.

The primers were designed and synthesized by Yixin (Wuxi, China) depending on the *AusM* sequence and the codons encoding mutation amino-acid residues. The site-directed mutagenesis of AusM was performed using the two-step PCR method. In detal, using recombinant plasmid pET-28a-*AusM* as a template, the first-round PCR was performed with a forward primer, such as F17Y-F and a reverse one (AusM-R) (Table 1) under the following conditions: initial denaturation at 98 °C for 3 min, followed by 35 cycles of at 98 °C for 10 s, 60 °C for 20 s and 72 °C for 25 s, and an additional elongation 72 °C for 10 min. Subsequently, the second-round PCR was carried out using pET-28a-*AusM* as a template, AusM-F and the first-round PCR’s product as forward and reverse primers, respectively: 35 cycles at 98 °C for 10 s, 60 °C for 20 s and 72 °C for 30 s, and an additional elongation 72 °C for 10 min. Finally, 2 μL target products purified by FastPure^®^ Gel Extraction Mini Kit and 3 μL forementioned linearized vector were incubated with 5 μL 2×Hieff Clone^®^ Enzyme Premix at 50 °C for 20 min and transformed into *E. coli* BL21(DE3), respectively, thereby producing fifteen *E. coli* transformants, including *E. coli*/*AusM*^F18H^, *E. coli*/*AusM*^N127A^ and *E. coli*/*AusM*^PKK^.

Single colonies of *E. coli* transformants, such as *E. coli*/*AusM*, *E. coli*/*AusM*^PKK^, were inoculated separately into the 2.5 mL LB medium containing 100 μg/mL kanamycin and cultured at 37 °C and 220 rpm for 14–16 h as the seed culture. Subsequently, fresh LB medium was inoculated with 1.5% (*v*/*v*) seed culture and grown for 2–3 h until OD_600_ reached 0.6–0.8. After inducing with 0.2 mM IPTG at 20 °C for 10–12 h, *E. coli* transformant cells were harvested by centrifugation (10,000 rpm, 4 °C, 5 min) and stored at −20 °C. As a negative control, *E. coli*/pET-28a(+), a transformant containing the plasmid pET-28a(+), was used.

### 2.5. Purification of Xylanase Inhibitor SyXIP-I and Xylanases

In our earlier research, the *XIP-I* gene (namely *SyXIP-I*) from *T. aestivum*, was synthesized and successfully expressed in wild-type *P. pastoris* X33 via the pGAPZαA vector without a 6 ×His-tag at the protein’s N/C-terminal amino acid. The pure SyXIP-I protein was isolated through two chromatographic steps, as described previously [29], with a minor modification. SyXIP-I’s protein concentration was determined using the Bradford assay with bovine serum albumin as the standard, and its purity was confirmed using SDS-PAGE. All purification steps were conducted at 4 °C unless otherwise stated.

For the purification of xylanases (AusM and its mutants), the induced *E. coli* cells were collected and resuspended in a lysis buffer containing 50 mM NaH_2_PO_4_, 500 mM NaCl, and 20 mM imidazole at pH 8.0. The suspension was adjusted to a fixed concentration of 100 mg wet cells/mL, unless otherwise stated. Subsequently, the cells were disrupted through sonication in an ice-water bath. The affinity chromatography purification process was carried out using a Co-nitrilotriacetic acid (Co-NTA) column Tiandz (Beijing, China). The concentration and purity of the enzymes were evaluated in the same ways.

For purification of the mixture of inhibitor and enzyme, 4 mg of the purified AusM and its mutants were individually incubated with an excess amount of purified SyXIP-I, at 30 °C for 30 min in 50 mM Na_2_HPO_4_-KH_2_PO_4_ buffer (pH 7.0). Following incubation, the mixture was purified according to the above method. The samples were then analyzed using SDS-PAGE. The inhibitor SyXIP-I and the xylanases were used as controls.

### 2.6. Xylanase Activity and Inhibition Assays

Xylanase activity was measured according to previous methods [28], with a slight modification. In summary, 20 μL of appropriately diluted xylanase enzyme sample was introduced into a solution containing 480 μL of 0.5% (*w*/*v*) birchwood xylan substrate dissolved in Citrate phosphate buffe (50 mM citric acid/50 mM Na_2_HPO_4_, pH 4.6) and the mixture was incubated at 50 °C for 15 min. The reaction was terminated by adding 500 μL of DNS reagent and boiling for 7 min. After the sample was cooled to room temperature, 1 mL of distilled water was added, and then the sample was evenly mixed. Subsequently, 200 μL of the mixture was transferred to a microtiter plate, and the absorbance at 540 nm was measured in relation to a xylose standard curve (0–200 ug/mL). Assays were repeated in triplicate. One unit (U) of xylanase activity was defined as the amount of enzyme releasing 1 μmol reducing sugar equivalent per minute under the given experiment conditions (pH 4.6 and 50 °C for 15 min).

An inhibition reaction was conducted in a final volume of 500 μL by combining 480 μL 0.5% (*w*/*v*) birchwood xylan, 0.055–0.065 U xylanase and a dilution of purified SyXIP-I. The ratios of SyXIP-I to xylanase were varied in the experiments. The residual xylanase activity was measured using the same conditions as described previously. The same buffer but without the inhibitor served as a negative control. The inhibition sensitivity was expressed as a percentage decrease in xylanase activity. The experiments were conducted in triplicate.

### 2.7. Effect of Temperature on the Activity and Stability of Xylanses

To investigate the influence of temperature on xylanase activity, the temperature optima for AusM and its best mutant AusM^PKK^ were measured using the aforementioned assay conditions [28], except for varying temperatures ranging from 45 °C to 80 °C. To assess the temperature stabilities of AusM and AusM^PKK^, both enzymes were incubated in 50 mM Na_2_HPO_4_-KH_2_PO_4_ buffer at pH 7.0 in the absence of substrate for 1.0 h at temperatures ranging from 45 °C to 80 °C, respectively. After the incubation, the temperature of the enzyme solution was cooled to 4 °C, and the residual enzyme activity was then measured. The experiments were conducted in triplicate.

### 2.8. Effect of EDTA and Metal Ions

The effect of EDTA and metal ions on the activity of purified xylanases (AusM and AusM^PKK^) was determined by incubating the enzyme with various metal ions and EDTA at a final concentration of 5 mM. The metal ions tested included Sn^2+^, Fe^2+^, Fe^3+^, Mg^2+^, Li^2+^, Ca^2+^, Cu^2+^, Zn^2+^, Ba^2+^ and Mn^2+^. The mixtures were incubated at 25 °C for 1.0 h, and the relative activity was determined by comparing it to the activity of the mixture without the addition of any metal ions or EDTA. The experiments were conducted in triplicate.

### 2.9. Determination of Kinetic Parameters

The kinetic parameters of the purified enzyme were calculated under the above xylanse activity assay method, except for concentrations of beechwood xylan (1.0–10 mg/mL). The apparent *K*_m_ and *V*_max_ values were calculated by non-linear regression analysis using Origin 9.0 software (http://www.originlab.com/, accessed on 1 August 2023). The experiments were conducted in triplicate.

### 2.10. Bread-Making Trials

Wheat bread (WB) was made using a recipe based on the method described [30], with a minor modification. The ingredients used in the recipe were as follows: 300 g of wheat flour, 4.5 g of yeast, 24 g of sugar, 3.0 g of salt, 24 g of butter, 179 g of tap water, and 1.0 mL of enzyme solution. Two types of xylanases, AusM and AusM^PKK^, were added for bread baking, with varying concentrations: AusM ranging from 0 to 5000 U/kg wheat flour and AusM^PKK^ from 0 to 1250 U/kg wheat flour. For the negative control, 1.0 mL of tap water was substituted for the enzyme solution. The process for baking bread was as follows: Firstly, all of the ingredients, excluding the butter, were mixed with a kneader (SM-25, Sinmag Equipment Crop., Wuxi, China) for 3 min at slow speed and followed by 1 min at high speed. The butter was then added, followed by a kneading process lasting 3 min at a low speed and an additional 3.4 min at a high speed. After resting for 5 min at room temperature, the dough was divided into 90 g pieces for molding, proofed at 38 °C and 80% relative humidity for 90 min, and then baked in an electric oven (Sinmag, SM-503+1S) at 170 °C (top) and 210 °C (bottom) for 20 min. After the freshly baked bread was cooled to room temperature for approximately 2 h, it was enclosed within a polyethylene bag and stored at 4 °C and 46% relative humidity for further analysis.

### 2.11. Specific Volume and Texture of Bread Assays

The specific volume of bread was determined using the rapeseed replacement method stated in AACC, 10-05 [31]. The specific volume was calculated by dividing the bread’s apparent volume (V) by its mass (m). The hardness of the bread was evaluated using a texture analyzer TA-XT2i (Stable Micro Systems, Godalming, Surrey, UK). The breads were cut into 12 mm thick slices using a bread slicing machine after being stored for 0, 1, 3, 5, and 7 days, respectively. For the hardness test, the middle two slices of at least three loaves were stacked together as a sample.

### 2.12. Statistical Analysis

Data was compared using one-way analysis of variance (ANOVA), while multiple comparisons of data were performed by Duncan’s test at *p* < 0.05 level of significance using SPSS 24.0. All data from three independent replicates were expressed as the mean ± standard deviation.

## 3. Results and Discussion

### 3.1. The Prediction of AusM-SyXIP Binding Sites

**Figure 1:**
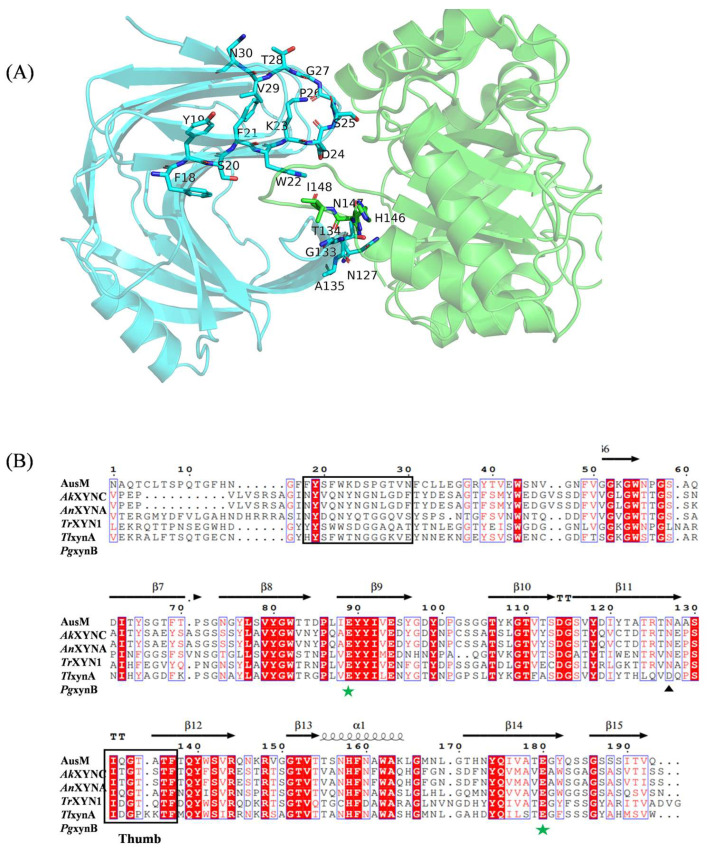
Rational design of AusM for its site-directed mutagenesis. (**A**) The 3-D structure of the docked AusM-SyXIP-I complex. SyXIP-I (**left side**) and AusM (**right side**) were shown. The selected residue sites for the site-directed mutagenesis of AusM were coloured in cyan. Residues, which may bind to AusM, were marked in green. (**B**) Multiple sequence alignment of AusM with other five selected GHF11 xylanases. *Ak*XYNC (GenBank: AAC60542); *Au*XYNA (GenBank: A19535); *Tr*XYN1 (GenBank: X69574); *Tl*xynA (GenBank: TLU35436); *Pg*xynB (GenBank: 63703464). The two catalytic residues were marked with green stars. The selected residue sites were marked with black boxes.

Until now, only one cocrystal structure of the XIP-I-XynC complex has been solved [32]. To obtain the AusM mutant with thermostability and insensitivity to SyXIP-I, a comprehensive mutational analysis was conducted using homology models and protein-protein docking of both AusM and SyXIP-I. As shown in Figure 1A, the 3D model of AusM-SyXIP complex was produced, in which a Π-shaped long loop from SyXIP-I protrudes between the thumb and palm of the enzyme, which agrees with the solved cocrystal structure (PDB: 1TEA). Interactions between SyXIP-I and specific regions surrounding the entrance of the active site groove in AusM are crucial for the recognition and binding of the inhibitor. Particularly, the thumb region played a significant role in the binding of SyXIP-I. Based on sequence alignments and the analysis of the above interactions with regions (thumb and palm), fifteen mutants, fourteen single-site and a three-amino acid replacement in the thumb region, namely AusM^F18H^, AusM^Y19A^, AusM^S20A^, AusM^F21A^, AusM^W22A^, AusM^K23T^, AusM^D24A^, AusM^S25A^, AusM^P26A^, AusM^G27A^, AusM^T28K^, AusM^V29A^, AusM^N30E^, AusM^N127A^, and AusM^PKK^, were designed and introduced via site-directed mutagenesis (Figure 1B). All mutants were expressed in *E. coli.*

### 3.2. Inhibitor Sensitivity Assay

**Figure 2:**
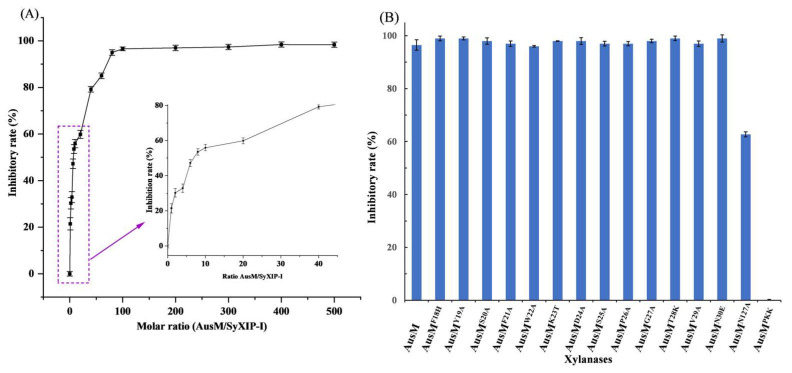
The effect of the inhibitory on xylanase activity. (**A**), the ratios between AusM and xylanase-inhibiting protein SyXIP-I were expressed as molar ratios. The right graph representeds a zoom of low relative ratios as it is unclear in the left graph; (**B**), Inhibitor sensitivity assays of xylanases of AusM and its fifteen mutants (AusM^F18H^, AusM^Y19A^, AusM^S20A^, AusM^F21A^, AusM^W22A^, AusM^K23T^, AusM^D24A^, AusM^S25A^, AusM^P26A^, AusM^G27A^, AusM^T28K^, AusM^V29A^ and AusM^N30E^, AusM^N127A^ and AusM^PKK^), were measured using a molar ratio of SyXIP-I/AusM of 100:1. Means and standard deviations of triplicate experiments are shown.

For the inhibitor sensitivity assay to SyXIP-I, both purified AusM and its mutants were used. The residual enzyme activity was measured by varying the molar ratio of inhibitor SyXIP-I to xylanase AusM (M/M). When the inhibition rate of AusM reached 50%, a molar ratio of 7:1 (SyXIP-I to AusM) was required, whereas more than 80 times the amount of SyXIP-I relative to AusM was necessary to achieve almost complete inhibition (Figure 2A). Therefore, using a higher inhibition addition at a molar ratio of SyXIP-I/xylanase of 100:1 as the criteria, the activity of fifteen mutants was measured respectively. The results showed that mutants AusM^F18H^, AusM^Y19A^, AusM^S20A^, AusM^F21A^, AusM^W22A^, AusM^K23T^, AusM^D24A^, AusM^S25A^, AusM^P26A^, AusM^G27A^, AusM^T28K^, AusM^V29A^ and AusM^N30E^ were completely inactivated by the inhibitor SyXIP-I, while AusM^N127A^ remained 62.7% of its initial activity and AusM^PKK^ remained its initial activity with no activity loss. (Figure 2B). The xylanase activity of the mutant AusM^PKK^ remained unaffected by the addition of SyXIP-I even up to a molar ratio of 200–500:1 (inhibitor:enzyme), as compared with 64.15–88.8% inhibition for the mutant AusM^N127A^. Compared to other methods reported in the literature, which were measured after xylanase was incubated with the purified inhibitor for 30 min [29,33], the method of this study was more suitable. Although SyXIP-I and xylanase bind quickly, it’s possible that adding them at the same time would require a higher concentration of SyXIP-I to achieve the same level of inhibition. Based on the above experiment, we expect that AusM is almost completely inhibited at low dosages during bread making (<2000 U/kg), while AusM^PKK^ activity is reduced by less than 20%.

### 3.3. Interaction of AusM and Its Best Mutant and with Inhibitor SyXIP-I

**Figure 3:**
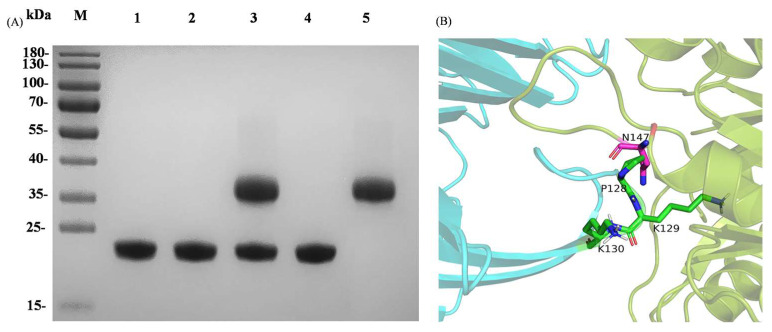
Analysis of the source of AusM^PKK^ with reduced inhibitor sensitivity for SyXIP-I. (**A**), SDS-PAGE analysis of the expressed xylanases and SyXIP-I, showing the interaction between xylanases and SyXIP-I. Lines 1 and 2, the purified xylanases AusM and its best mutant AusM^PKK^. Lines 3 and 4, the purified AusM and its best mutant incubated with an excess amount of purified SyXIP-I for 30 min at 30 °C, line 5, the purified SyXIP-I. (**B**), The 3-D structure of the aligned docked AusM^PKK^-SyXIP complex. The residue sites where interactions were probable were marked in green and purple.

A stop codon was inserted at the C-terminus of the *Syxip-I* gene in the recombinant plasmid, resulting in the absence of a His-tag in the expressed protein. AusM, along with its mutants, incubated with an excess amount of purified SyXIP-I, respectively, for 30 min at 30 °C, were further purified by affinity chromatography utilizing a Co-nitrilotriacetic acid (Co-NTA) column. To determine whether the loss of inhibition measured in activity assays coincided with the interaction between xylanase and SyXIP-I, xylanases AusM and its best mutant were tested via binding assays without xylan substrate. The purified proteins were subsequently subjected to SDS-PAGE analysis. The purified xylanase AusM and its best mutant AusM^PKK^ and SyXIP-I exhibited distinct single bands on the SDS-PAGE gel, with approximate molecular masses of 22 kDa and 34 kDa, respectively, aligning with their expected molecular weights (Figure 3A, lines 1–2, 5). However, for xylanase AusM, two bands were observed on the SDS-PAGE gel. One band corresponded to the xylanase, while the other corresponded to SyXIP-I (Figure 3A, line 3).

In contrast, the best mutant AusM^PKK^ displayed only a single band (Figure 3A, line 4), consistent with the result from the activity assay in Figure 2B. To gain insight into the source of AusM^PKK^ with reduced inhibitor sensitivity, the 3-D conformation of docked was compared with that of AusM-SyXIP-I. In the model AusM^PKK^-SyXIP-I complex, strong steric clashes are observed between P128 in AusM^PKK^ and N147 in SyXIP, which would prevent XIP from entering the catalytic binding pocket of AusM^PKK^, thereby not affecting the AusM^PKK^’s catalytic reaction on the substrate.

### 3.4. Effect of Temperature on the Activity and Stability of Xylanases

**Figure 4:**
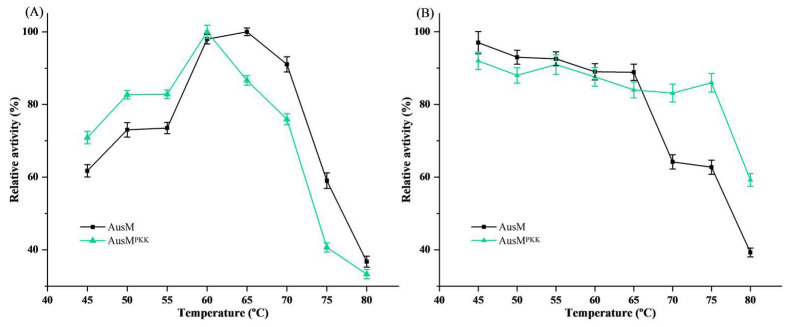
The optimum temperature and thermostability of AusM and AusM^PKK^. (**A**) The optimum temperature of AusM and AusM^PKK^; (**B**) The thermostability of AusM and AusM^PKK^. The AusM and AusM^PKK^ were incubated in 50 mM Na_2_HPO_4_-KH_2_PO_4_ buffer (pH 7.0) at different temperatures (45 °C to 80 °C) for 1.0 h. Means and standard deviations of triplicate experiments are shown.

The effect of temperature on enzyme activity and the stability of AusM and AusM^PKK^ were determined across a wide range of temperatures. As depicted in Figure 4A, the temperature profiles of enzyme activity were illustrated. AusM displayed its maximum enzyme activity at 60–65 °C, while AusM^PKK^ exhibited its highest activity at 60 °C. Remarkably, even at 70 °C, both enzymes retained over 90% and 75% of their maximal enzyme activity, respectively. Notably, their optimal temperature exceeded those typically observed for fungal xylanases, which generally work between 45 °C and 55 °C. [34,35,36]. Consequently, AusM and AusM^PKK^ demonstrate a broader operational temperature range and enhanced efficiency in xylan hydrolysis at elevated temperatures. As shown in Figure 4B, when AusM and AusM^PKK^ were incubated in 50 mM phosphate buffer (pH 7.0) at a temperature range of 45 °C to 80 °C for 60 min, respectively, AusM showed considerably high enzyme activity (>60%) at temperatures 45 °C to 75 °C, while AusM^PKK^ displayed superior thermostability with relative enzyme activity exceeding 85% within the same temperature range, and even retained approximately 60% of its xylanase activity at 80 °C. The two enzymes showed excellent thermostability characteristics compared to most reported enzymes (Table S1). For instance, *Penicillium oxalicum* GZ-2 produced XYN11A, an acidophilic xylanase, which lost 86% of its activity after 15 min at 55 °C [37]. Similarly, the recombinant xylanase from *Myceliophthora heterothallica* displayed approximately 30% residual activity following a 60-min incubation at 70 °C [38]. Considering the context of the bread-baking industry, where wheat starches typically start gelatinizing at 60 °C or higher, high optimal temperature, remarkable thermostability, and insensitivity to SyXIP-I indicate that AusM^PKK^ has a distinct advantage for bread-baking applications.

### 3.5. Enzymatic Properties of the Purified AusM and AusM^PKK^

**Figure 5:**
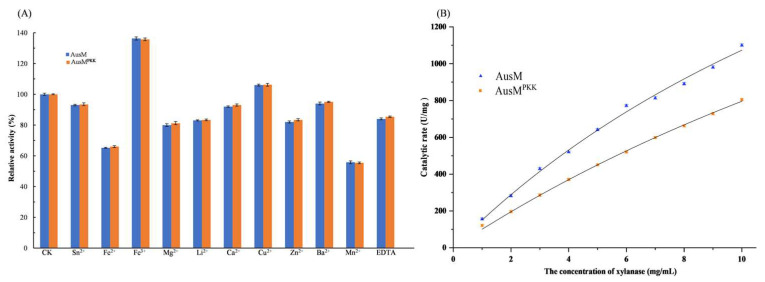
Enzymatic properties of the purified AusM and AusM^PKK^. (**A**), Effects of EDTA and various metal ions on the activity of AusM and AusM^PKK^. (**B**), The enzymatic kinetic assay results plotted for purified AusM and AusM^PKK^.

Metal ions play crucial roles in the catalytic reaction of the enzyme, particularly as electron acceptors or donors. In this study, the purified xylanases, AusM and AusM^PKK^, were incubated with various metal ions and EDTA at 25 °C for 1.0 h, their enzyme activities were not affected (100 ± 5%) by Sn^2+^ Mg^2+^, Li^2+^, Ca^2+^, Cu^2+^, Zn^2+^, Ba^2+^, Mn^2+^ and EDTA, while they were strongly increased Fe^3+^ (136.3% for AusM and 135.7% for AusM^PKK^) and inhibited by Fe^2+^ (65.2% for AusM and 66.0% for AusM^PKK^) and Mn^2+^ (55.8% for AusM and 55.4% for AusM^PKK^) (Figure 5A). One potential explanation for the observed stimulation of xylanase activity by Fe^3+^ may be its influence on the bonding and decomposition state of the enzyme-substrate complex. On the contrary, thiol groups of cysteine residues in the active sites or nearby appear responsible for the inhibition. It is possible that the inhibition of xylanase by Fe^2+^ and Mn^2+^ is also due to the formation of ion-enzyme complexes at the active site [39].

The *K*_m_ and *V*_max_ values of the purified AusM and AusM^PKK^ towards birchwood xylan were graphically determined by using Origin non-linear curve fitting as shown in Figure 5B to be 21.31 mg/mL and 3360.87 U/mg for AusM and 32.96 mg/mL and 3419.45 U/mg, respectively. The *V*_max_ values of AusM and AusM^PKK^ were unchanged before and after the mutation, which was comparable to those previously reported in the range of 400–5000 U/mg while *K*_m_ is relatively higher than those previously reported in the range of 4–5 mg/mL [21,40]. The *K*_m_ value of AusM^PKK^ was relatively higher than that of AusM, indicating that the affinity of AusM^PKK^ for the xylan substrate was less than that of AusM. Similarly, the specific activity of AusM (1100.82 U/mg) was higher than that of AusM^PKK^ (975.38 U/mg)

### 3.6. Effect of Xylanase on Specific Bread Loaf Volume

**Figure 6:**
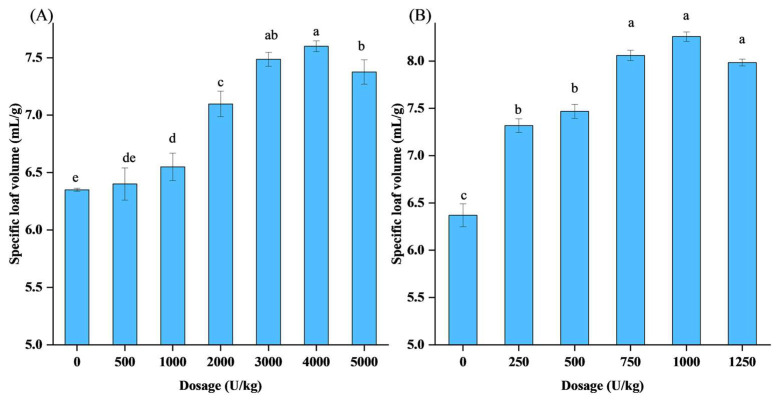
The effect of xylanase on specific bread loaf volume. (**A**), comparison of seven groups’ bread loaf volume. Groups with 0, 500, 1000, 2000, 3000, 4000, 5000 U/kg AusM, respectively. (**B**), comparison of six groups’ bread loaf volume. Groups with 0, 250, 500, 1000, 1250 U/kg AusM^PKK^, respectively. Means and standard deviations of triplicate experiments are shown. Different letters indicate significant differences (*p* < 0.05).

Figure 6 illustrates the relationship between the xylanase dosage and the corresponding increase in specific loaf volume. Using a range of AusM concentrations (500, 1000, 2000, 3000, 4000, and 5000 U/kg), as well as AusM^PKK^ concentrations (250, 500, 750, 1000, and 1250 U/kg), and a control containing no xylanase, the effects of AusM and the mutant AusM^PKK^ on bread quality were compared. At doses ranging from 0 to 1000 U/kg, the specific loaf volume did not differ significantly when AusM was added to bread, indicating that AusM may be inactivated by the inhibitor in the wheat flour. However, a range of dosages (3000–5000 U/kg) was required to achieve a significantly higher increase in specific loaf volume, with the maximum increase in specific loaf volume (19.7%) obtained at a dosage of 4000 U/kg (Figure 6A). For the resistant xylanase AusM^PKK^, the range of dosages was maintained within a narrower range of 250–1250 U/kg. At a dosage of 1000 U/kg, the specific loaf volume increased by 26.5% compared to a control bread sample with no xylanase added (Figure 6B). Comparatively, four times as much AusM as AusM^PKK^ was required to counteract the inhibition effect of XIP-I and achieve an equivalent impact on specific loaf volume.

Prior studies have demonstrated that the incorporation of xylanase has the potential to substantially enhance various properties of wheat flour dough, including water absorption, development time, stability, stickiness, and mixing tolerance index (MTI) [41]. Conversely, adding xylanase to wheat bran containing 15% bran did not yield significant differences in water absorption, development time, stability, and departure time for wheat bran dough [9]. These distinctions are plausibly attributed to XIP-I originating from the aleurone-enriched fraction in wheat bran, influencing the activity of xylanase. The aleurone-enriched fraction, comprising approximately 7% of the caryopsis weight, accounted for 18.2% of the total XIP content [19]. Given the above, AusM^PKK^ with insensitivity to SyXIP-I, has excellent potential for application as an additive in the bread industry.

### 3.7. Effect of Xylanase on the Texture of Bread during Storage

**Figure 7:**
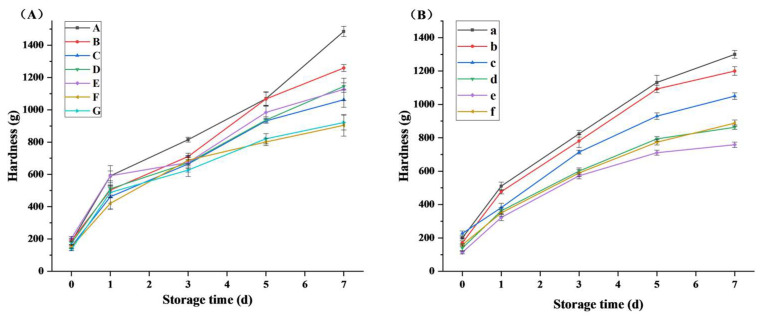
The effect of xylanase on the texture of bread. (**A**), comparison of seven groups’ bread hardness during storage. Groups A–G, with 0, 500, 1000, 2000, 3000, 4000, 5000 U/kg AusM, respectively. (**B**), comparison of six groups’ bread loaf volume. Groups a–f with 0, 250, 500, 1000, 1250 U/kg AusM^PKK^, respectively.

The hardness of bread is correlated with a deterioration in bread-making quality. Figure 5 shows the effects of AusM and AusM^PKK^ on bread hardness during storage. As the storage duration progressed to 5 and 7 days, a gradual rise in bread hardness was observed, compared to the control groups A and a, both AusM and AusM^PKK^ exhibited pronounced effects in decreasing bread hardness. After seven days of storage, the AusM (2000–5000 U/kg) significantly reduced the hardness of the bread in groups D, E, F, and G by 22.9–39.16%, respectively (Figure 7A). Similarly, AusM^PKK^ (500–1000 U/kg) was able to significantly decrease the hardness of bread (Figure 7B). As storage time increased, the difference between group e (1000 U/kg) and group a (control) became more pronounced, demonstrating that AusM^PKK^ retarded the decline in bread hardness. Additionally, when comparing AusM and AusM^PKK^ at their respective optimal dosages (AusM 4000 U/kg and AusM^PKK^ 1000 U/kg), there was a significant difference between the breads from day 0 to day 7 of storage. The bread treated with 1000 U/kg AusM^PKK^ exhibited lower hardness compared to that treated with 4000 U/kg AusM by 11.2% and 16.05%, respectively (day three and day 7), indicating the insensitivity AusM^PKK^ had a better effect on reducing bread hardness than sensitivity AusM.

## 4. Conclusions

Xylanase usually requires high enzyme activity and a high-temperature range for activity to exert the desired effects in bread making. Here, based on the computer-aided re-design of AusM, AusM was evolved to mutant AusM^PKK^. The mutant AusM^PKK^ showed significantly improved insensitivity compared with AusM towards SyXIP-I, and an optimum temperature of 60 °C, slightly lower than that of AusM. The relative affinities of the interactions between AusM or its best mutant AusM^PKK^ and SyXIP-I were studied using affinity chromatography and visually shown on SDS-PAGE. In bread making, 1000 U/kg AusM^PKK^ showed a better effect on specific bread loaf volume than 4000 U/kg AusM. Even after seven days of storage, 1000 U/kg AusM^PKK^ decreased bread hardness to 4000 U/kg AusM. Thus, mutant AusM^PKK^ with insensitivity is more conducive to extending the shelf life of bread, and it can be applied at a significantly lower dose than the original enzyme. AusM^PKK^ is an excellent, potentially economical, valuable xylanase suitable for application as an additive in the bread-making industry.

## Figures and Tables

**Table 1:** PCR primers used in this work. (The codons encoding mutation residues are underlined).

Primer Name	Primer Sequence (5′→3′)
AusM-F	CTTTAAGAAGGAGATATACCATGAACGCTCAAACTTGT
AusM-R	GTGGTGGTGGTGGTGGTGCTGAACAGTGATGGACGAAGA
r-pET-28a-F	CATGGTATATCTCCTTCTTAAAG
r-pET-28a-R	CACCACCACCACCACCAC
F18H-F	TCACAACGGTTTCCACTACTCTTTCTGGAAG
Y19A-F	AACGGTTTCTTCGCGTCTTTCTGGAAG
S20A-F	GGTTTCTTCTACGCGTTCTGGAAGGAC
F21A-F	TTCTTCTACTCTGCGTGGAAGGACAGT
W22A-F	TTCTACTCTTTCGCGAAGGACAGTCCA
K23T-F	TTCTACTCTTTCTGGACCGACAGTCCAGGT
D24A-F	TCTTTCTGGAAGGCGAGTCCAGGTACT
S25A-F	TTCTGGAAGGACGCGCCAGGTACTGTT
P26A-F G27A-F T28K-F V29A-F N30E-F N127A-F	CTGGAAGGACAGTGCGGGTACTGTTAATTTT AAGGACAGTCCAGCGACTGTTAATTTT GACAGTCCAGGTAAGGTTAATTTTTGT AGTCCAGGTACTGCGAATTTTTGTCTG CCAGGTACTGTTGAGTTTTGTCTGTTG CGGCTACCCGTACCAATGCGGCTTCCATTCA
PKK-F	GCTGCTTCCATTCAGGGACCAAAGAAGACCTTCACTCAGTAC

## Data Availability

Data is contained within the article or supplementary material.

## Supplementary Materials

The following supporting information can be downloaded at https://www.mdpi.com/article/10.3390/foods12193574/s1, see refs [42,43,44,45], Table S1: The preparation of optimal temperature and thermostability of various enzymes.

## References

[1] Leys S., Bondt Y.D., Bosmans G., Courtin C.M. (2020). Assessing the impact of xylanase activity on the water distribution in wheat dough: A H^1^ NMR study. Food Chem..

[2] Xue Y.M., Cui X.B., Zhang Z.H., Zhou T., Gao R., Li Y.X., Ding X.X. (2019). Effect of β-endoxylanase and α-arabinofuranosidase enzymatic hydrolysis on nutritional and technological properties of wheat brans. Food Chem..

[3] Leys S., Bondt Y.D., Schreurs L., Courtin C.M. (2019). Sensitivity of the *Bacillus subtilis* Xyn A xylanase and its mutants to different xylanase inhibitors determines their activity profile and functionality during bread making. J. Agric. Food Chem..

[4] Carvalho E.A., dos Santos Góes L.M.D., Uetanabaro A.P.T., da Silva E.G.P., Rodrigues L.B., Pirovani C.P., da Costa A.M. (2017). Thermoresistant xylanases from *Trichoderma stromaticum*: Application in bread making and manufacturing xylo-oligosaccharides. Food Chem..

[5] Ktenioudaki A., Gallagher E. (2012). Recent advances in the development of high-fibre baked products. Trends Food Sci. Tech..

[6] Ordaz-Ortiz J.J., Saulnier L. (2005). Structural variability of arabinoxylans from wheat flour. Comparison of water-extractable and xylanase-extractable arabinoxylans. J. Cereal Sci..

[7] Izydorczyk M.S., Biliaderis C.G. (1995). Cereal arabinoxylans: Advances in structure and physicochemical properties. Carbohyd. Polym..

[8] Courtin C.M., Roelants A., Delcour J.A. (1999). Fractionation-reconstitution experiments provide insight into the role of endoxylanases in bread-making. J. Agric. Food Chem..

[9] Liu W.J., Brennan M.A., Serventi L., Brennan C.S. (2017). Effect of cellulase, xylanase and α-amylase combinations on the rheological properties of Chinese steamed bread dough enriched in wheat bran. Food Chem..

[10] Oliveira D.S., Telis-Romero J., Da-Silva R., Franco C.M.L. (2014). Effect of a *Thermoascus aurantiacus* thermostable enzyme cocktail on wheat bread qualitiy. Food Chem..

[11] Collins T., Gerday C., Feller G. (2005). Xylanases, xylanase families and extremophilic xylanases. FEMS Microbiol. Rev..

[12] Driss D., Berrin J.G., Juge N., Bhiri F., Ghorbel R., Chaabouni S.E. (2013). Functional characterization of *Penicillium occitanis* Pol6 and *Penicillium funiculosum* GH11 xylanases. Protein Expr. Purif..

[13] Courtin C.M., Delcour J.A. (2001). Relative activity of endoxylanases towards water-extractable and water-unextractable arabinoxylan. J. Cereal Sci..

[14] Chadha B.S., Kaur B., Basotra N., Tsang A., Pandey A. (2019). Thermostable xylanases from thermophilic fungi and bacteria: Current perspective. Bioresour. Technol..

[15] Rouau X., Daviet S., Tahir T., Cherel B., Saulnier L. (2006). Effect of the proteinaceous wheat xylanase inhibitor XIP-I on the performances of an *Aspergillus niger* xylanase in breadmaking. J. Sci. Food Agric..

[16] Gebruers K., Brijs K., Courtin C.M., Fierens K., Goesaert H., Rabijns A., Raedschelders G., Robben J., Sansen S., Sørensen J.F. (2004). Properties of TAXI-type endoxylanase inhibitors. BBA Proteins Proteom..

[17] Juge N., Payan F., Williamson G. (2004). XIP-I, a xylanase inhibitor protein from wheat: A novel protein function. BBA Proteins Proteom..

[18] Fierens E., Gebruers K., Voet A.R.D., De Maeyer M., Courtin C.M., Delcour J.A. (2009). Biochemical and structural characterization of TLXI, the *Triticum aestivum* L. thaumatin-like xylanase inhibitor. J. Enzym. Inhib. Med. Chem..

[19] Croes E., Gebruers K., Luyten N., Delcour J.A., Courtin C.M. (2009). Immunoblot quantification of three classes of proteinaceous xylanase inhibitors in different wheat (*Triticum aestivum*) cultivars and milling fractions. J. Agric. Food Chem..

[20] Berrin J.G., Juge N. (2008). Factors affecting xylanase functionality in the degradation of arabinoxylans. Biotechnol. Lett..

[21] Zhu D.D., Liu X.Y., Xie X., Yang S., Lin H., Chen H.G. (2020). Characteristics of a XIP-resistant xylanase from *Neocallimastix* sp. GMLF 1 and its advantage in barley malt saccharification. Int. J. Food Sci. Tech..

[22] Sancho A.I., Faulds C.B., Svensson B., Bartolomé B., Williamson G., Juge N. (2003). Cross-inhibitory activity of cereal protein inhibitors against α-amylases and xylanases. BBA Proteins Proteom..

[23] Ito K., Iwashita K., Iwano K. (1992). Cloning and sequencing of the xynC gene encoding acid xylanase of *Aspergillus kawachii*. Biosci. Biotech. Bioch..

[24] Sansen S., De Ranter C.J., Gebruers K., Brijs K., Courtin C.M., Delcour J.A., Rabijns A. (2004). Structural basis for inhibition of *Aspergillus niger* xylanase by *Triticum aestivum* xylanase inhibitor-I. J. Biol. Chem..

[25] Törrönen A., Mach R.L., Messner R., Gonzalez R., Kalkkinen N., Harkki A., Kubicek C.P. (1992). The two major xylanases from *Trichoderma reesei*: Characterization of both enzymes and genes. Nat. Biotechnol..

[26] Schlacher A., Holzmann K., Hayn M., Steiner W., Schwab H. (1996). Cloning and characterization of the gene for the thermostable xylanase XynA from *Thermomyces lanuginosus*. J. Biotechnol..

[27] Tison M.C., Andre-Leroux G., Lafond M., Georis J., Juge N., Berrin J.G. (2009). Molecular determinants of substrate and inhibitor specificities of the *Penicillium griseofulvum* family 11 xylanases. BBA Proteins Proteom..

[28] Zhang H.M., Li J.F., Wang J.Q., Yang Y.J., Wu M.C. (2014). Determinants for the improved thermostability of a mesophilic family 11 xylanase predicted by computational methods. Biotechnol. Biofuels.

[29] Fierens K., Geudens N., Brijs K., Courtin C.M., Gebruers K., Robben J., Campenhout S.V., Volckaert G., Delcour J.A. (2004). High-level expression, purification, and characterization of recombinant wheat xylanase inhibitor TAXI-I secreted by the yeast *Pichia pastoris*. Protein Expr. Purif..

[30] Zhang B.L., Yang Z.X., Huang W.N., Omedi J.O., Wang F., Zou Q.B., Zheng J.X. (2019). Isoflavone aglycones enrichment in soybean sourdough bread fermented by lactic acid bacteria strains isolated from traditional Qu starters: Effects on in vitro gastrointestinal digestion, nutritional, and baking properties. Cereal Chem..

[31] AACC (2000). Approved Methods of the American Association of Cereal Chemists.

[32] Payan F., Leone P., Porciero S., Furniss C., Tahir T., Williamson G., Durand A., Manzanares P., Gilbert H.J., Juge N. (2004). The dual nature of the wheat xylanase protein inhibitor XIP-I: Structural basis for the inhibition of family 10 and family 11 xylanases. J. Biol. Chem..

[33] Belien T., Campenhout S.V., Acker M.V., Robben J., Courtin C.M., Delcour J.A., Volckaert G. (2007). Mutational analysis of endoxylanases XylA and XylB from the phytopathogen *Fusarium graminearum* reveals comprehensive insights into their inhibitor insensitivity. Appl. Environ. Microb..

[34] Dao T.M.A., Cuong N.T., Nguyen T.T., Nguyen N.P.D., Tuyen D.T. (2022). Purification, identification, and characterization of a glycoside hydrolase family 11-xylanase with high activity from *Aspergillus niger* VTCC 017. Mol. Biotechnol..

[35] Dutta T., Sengupta R., Sahoo R., Ray S.S., Bhattacharjee A., Ghosh S. (2007). A novel cellulase free alkaliphilic xylanase from alkali tolerant *Penicillium citrinum*: Production, purification and characterization. Lett. Appl. Microbiol..

[36] Silva L.A.O., Terrasan C.R.F., Carmona E.C. (2015). Purification and characterization of xylanases from *Trichoderma inhamatum*. Electron. J. Biotechnol..

[37] Liao H.P., Sun S.W., Wang P., Bi W.L., Tan S.Y., Wei Z., Mei X.L., Liu D.Y., Raza W., Shen Q.R. (2014). A new acidophilic endo-β-1,4-xylanase from *Penicillium oxalicum*: Cloning, purification, and insights into the influence of metal ions on xylanase activity. J. Ind. Microbiol. Biotechnol..

[38] de Amo G.S., Bezerra-Bussoli C., da Silva R.R., Kishi L.T., Ferreira H., Mariutti R.B., Arni R.K., Gomes E., Bonilla-Rodriguez G.O. (2019). Heterologous expression, purification and biochemical characterization of a new xylanase from *Myceliophthora heterothallica* F.2.1.4. Int. J. Biol. Macromol..

[39] Intasit R., Cheirsilp B., Suyotha W., Boonsawang P. (2022). Purification and characterization of a highly-stable fungal xylanase from *Aspergillus tubingensis* cultivated on palm wastes through combined solid-state and submerged fermentation. Prep. Biochem. Biotechnol..

[40] Silva P.O.D., Guimaraes N.C.D.A., Serpa J.D.M., Masui D.C., Marchetti C.R., Verbisck N.V., Zanoelo F.F., Ruller R., Giannesi G.C. (2019). Application of an endo-xylanase from *Aspergillus japonicus* in the fruit juice clarification and fruit peel waste hydrolysis. Biocatal. Agric. Biotech..

[41] Pescador-Piedra J.C., Garrido-Castro A., Chanona-Perez J., Farrera-Rebollo R., Gutierrez-Lopez G., Calderon-Dominguez G. (2009). Effect of the addition of mixtures of glucose oxidase, peroxidase and xylanase on rheological and breadmaking properties of wheat flour. Int. J. Food Prop..

[42] Li Y.Y., Li C., Huang H., Rao S.Q., Zhang Q., Zhou J.W., Li J.H., Du G.C., Liu L. (2022). Significantly enhanced thermostability of *Aspergillus niger* xylanase by modifying its highly flexible regions. J. Agr. Food Chem..

[43] Yang R., Li J.C., Teng C., Li X.T. (2016). Cloning, overexpression and characterization of a xylanase gene from a novel *Streptomyces rameus* L2001 in *Pichia pastoris*. J. Mol. Catal. B Enzym..

[44] Singh A., Sharma D., Varghese L.M., Mahajan R. (2020). Fast flow rate processes for purification of alkaline xylanase isoforms from *Bacillus pumilus* AJK and their biochemical characterization for industrial application purposes. Biotechnol. Progr..

[45] Liu M.Q., Weng X.Y., Sun J.Y. (2006). Expression of recombinant *Aspergillus niger* xylanase A in *Pichia pastoris* and its action on xylan. Protein Expr. Purif..

