# Supplementary material for: Directed Modification of a GHF11 Thermostable Xylanase AusM for Enhancing Inhibitory Resistance towards SyXIP-I and Application of AusMPKK in Bread Making
Source: Foods. 2023 Sep 26;12(19):3574. doi: 10.3390/foods12193574 (PMC10572589; doi:10.3390/foods12193574)
Supplement: Supplementary file 1 [file foods-12-03574-s001.zip › foods-2613140-supplementary.pdf]

Supplementary Data

Directed modification anti-inhibitory proteins of a GH11 thermostable xylanase AusM and its application in bread making

Dong Zhang1,2, Jing Huang3, Youyi Liu1, Xingyi Chen, Tiecheng Gao4, Ning Li4, Weining Huang3,*, Minchen Wu1,2,*

[1] Wuxi School of Medicine, Jiangnan University, Wuxi 214122, PR China;

[2] Key Laboratory of Carbohydrate Chemistry and Biotechnology, Ministry of Education, School of Biotechnology, Jiangnan University, Wuxi 214122, PR China;

[3] State Key Laboratory of Food Science and Technology, and the Laboratory of Baking and Fermentation Science, Cereals/Sourdough and Nutritional Functionality Research, Jiangnan University, Wuxi 214122, PR China;

[4] Guangzhou Puratos Food Co. Ltd., Guangzhou 511400, PR China

Dong Zhang and Jing Huang, the two first authors, contributed equally to this work

*Correspondences: wnhuang@jiangnan.edu.cn (W. N. Huang), biowmc@126.com (M. C. Wu)

Contents

Table S1 The preparation of optimal temperature and thermostability of various enzymes.

Enzyme	Optimal temperature (°C)	Residual activity (%)	The incubation temperature and time(min)	Reference
*An*xynA	50	50	50 °C, 18.2 min	[42]
*Sr*XynA	55	＞70	50 °C, 30 min	[43]
*Po*XYN11A	50	14	55 °C, 15 min	[37]
*Mh*xyl	55	30	70 °C, 60 min	[38]
*Bp*CX I-V	55	38-42	50 °C, 180 min	[44]
*An*xA	50	＞70	25 °C, 60 min	[45]
$AusM^{PKK}$	60	85.97	70 °C, 60 min	This study
